# Integrated metabolomic and transcriptomic analyses reveal molecular response of anthocyanins biosynthesis in perilla to light intensity

**DOI:** 10.3389/fpls.2022.976449

**Published:** 2022-09-23

**Authors:** Guanwen Xie, Xiuzai Zou, Zishan Liang, Duan Wu, Jiankuang He, Kaicheng Xie, Honglei Jin, Hongbin Wang, Qi Shen

**Affiliations:** Institute of Medical Plant Physiology and Ecology, School of Pharmaceutical Sciences, Guangzhou University of Chinese Medicine, Guangzhou, China

**Keywords:** *Perilla frutescens*, metabolomic, transcriptomic, photoinduced, weighted gene co-expression network analysis (WGCNA), transcription factor

## Abstract

The perilla anthocyanins have important medicinal and ornamental value, and their contents are significantly affected by light intensity. In view of their molecular mechanisms were not well understood, we integrated the metabolomic and transcriptomic analyses of the light-sensitive perilla variety under different light intensity. The perilla leave color were obviously affected under different treatments. Totally 140 flavonoid metabolites and 2461 genes showed steady change, among which 60 flavonoid metabolites were increased accumulation and 983 genes were upregulated expression under elevated light intensity treatment. Light treatment prominently affected the expression of genes involved in the main anthocyanin metabolites accumulation in perilla leaves. Using WGCNA analysis, we identified 4 key genes in anthocyanin biosynthesis pathway (*CHI*, *DFR*, and *ANS*) and 147 transcription factors (*MYB, bHLH, bZIP, ERF, and NAC*) involved in malonylshisonin biosynthesis. Among them, 6 MYBs and 4 bZIPs were predicted to play important roles in light regulation of malonylshisonin biosynthesis based on phylogenetic construction, correlation analysis, *cis*-acting element identification and qPCR verification. The identified key genes and regulatory factors will help us to understand the potential mechanism of photo-regulated anthocyanin accumulation in perilla.

## Introduction

Light is essential for plant growth and plays a key environmental factor regulating plant development. The intensity, quality, periodicity, and direction of light directly affect the biosynthesis of metabolites in plants. The influence of light on anthocyanin formation plays an important role in controlling the color of fruits such as grape (Azuma et al., 2012), eggplant (Jiang et al., 2016) and apple (Feng et al., 2013).

Anthocyanins, the largest group of natural water-soluble plant pigments, are widely present in flowers, fruits, leaves, and tubers in the plant kingdom (Chen et al., 2012). Anthocyanins are phenolic compounds containing C6-C3-C6 hydroxyl aromatic rings as a basic skeleton (Kong et al., 2003). The type and final color of anthocyanin are determined by its main backbone and the positions, quantities, and structures of conjugated sugars and covalent modifications (Kocic et al., 2011; Liu Y. et al., 2018). The major anthocyanins include cyanidin, delphinidin, malvidin, pelargonidin, peonidin, and petunidin, as well as their derivatives (Kong et al., 2003; Zhao et al., 2014; Khoo et al., 2017). Anthocyanins are important ingredients of healthy foods, with medicinal value for preventing neurological and cardiovascular diseases, cancer, diabetes, and age-related degenerative diseases (Kong et al., 2003; Zhao et al., 2014; Khoo et al., 2017). Hence, anthocyanins have attracted extensive attention worldwide in recent years.

*Perilla frutescens* (L.) is an annual herb in the Lamiaceae. Perilla has been widely cultivated in China for more than 2000 years and is currently cultivated in Korea, Japan, and other Asian countries (Nitta et al., 2005a). Perilla is used as traditional herb, oil crops and popular spice. This main medicinal value of perilla is listed in the Chinese Pharmacopeia, including anti-oxidant (Saita et al., 2012), antibacterial (Yamamoto and Ogawa, 2002), anti-inflammatory (Yang et al., 2013; Huang et al., 2014), cough-suppressing (Ahmed, 2019), lipid-lowering, and antitumor properties (Lin et al., 2007). Perilla is divided into red perilla and green perilla according to plant color. Besides its medicinal value, red perilla is also used as an ornamental plant due to its dark red leaves and stems. The red pigment of perilla leaves is also widely used as a food coloring, cosmetic additive, and medicinal compound.

Malonylshisonin [cyanidin 3-*O*-(6”-*O*-(E)-*p*-coumaryl)-β-D-glucopyranoside-5-*O*- (6”’-*O*-malonyl)-β-D-glucopyranoside], an unique and major cyanindin-type anthocyanin, was identified in red perilla (Honda et al., 1994; Saito and Yamazaki, 2002; Fujiwara et al., 2018; Jiang et al., 2020). They possessed antioxidant and anti-allergy properties in previous research (Makino et al., 2003; Meng et al., 2006). The biosynthetic pathway of malonylshisonin was recently elucidated. The basic anthocyanin steps involved in 4-coumaroyl-CoA were catalyzed to form cyanidin. The main steps were catalyzed by chalcone synthase (CHS), chalcone isomerase (CHI), flavanone 3-hydroxylase (F3H), flavonoid 3’-hydroxylase (F3’H), dihydroflavonol 4-reductase (DFR), and anthocyanin synthase (ANS). Whereafter, the cyanidin is converted to malonylshisonin via a process catalyzed by flavonoid 3-glucosyltransferase (3-GT), anthocyanin acyltransferase (ACT), anthocyanin 5-glucosyltransferase (5-GT), and anthocyanin malonyltransferase (MAT) (Saito and Yamazaki, 2002; Jiang et al., 2020; Zhou et al., 2021). As previous research in other plants, anthocyanin biosynthesis is regulated by the MBW ternary transcriptional complex (R2R3-MYB, basic helix-loop-helix [bHLH], and WD40 repeat protein [WDR]) (Holton and Cornish, 1995; Feng et al., 2010; Lloyd et al., 2017), basic leucine zipper (bZIP) (An et al., 2017), and MYC transcription factors.

Light could induce anthocyanin accumulation in perilla leaves. In a previous study of red perilla, anthocyanin accumulation and the expression of anthocyanin biosynthetic genes in leaves were lower under weak vs. strong light conditions (Zheng et al., 2019). However, the molecular mechanisms underlying light-induced anthocyanin biosynthesis in perilla remain unknown. Here, we explored anthocyanin biosynthesis and the mechanism of its photoinduction in red perilla using a light-sensitive cultivated variety. We treated the plants with different light intensities and analyzed the regulatory networks of anthocyanin biosynthesis and accumulation by performing comprehensive metabolomic and transcriptomic analyses. Our findings shed light on the putative molecular regulation of photoinduced anthocyanin accumulation in red perilla.

## Materials and methods

### Plant materials and treatments

In our previous work, we planted more perilla varieties in light incubator and found that the plant color of ‘PF899’ variety was more sensitive to light intensity. The variety is extremely sensitive to changes in light intensity, with green leaves in weak light and dark red leaves in increased light intensity. Hence, the light-sensitive perilla variety was selected in the study. After germination, the plants were grown in a greenhouse at the Guangzhou University of Chinese Medicine in Guangdong province, China (23°3’N, 113°23’E) under a weak light intensity (WL; 15 μmol/m^2^) until the three-leaf stage. The plants were transferred to moderate light (ML; 180 μmol/m^2^) for 6 days, followed by transfer to weak light (15 μmol/m^2^) for 6 days (recovery light conditions, RL). The plants were grown under a 12 h/12 h light/dark photoperiod and fertilized according to standard practices. Each treatment group was treated with three pots, each pot had five plants. Three biological replicates were performed for each treatment group. The leaves from three treatments were collected, immediately frozen in liquid nitrogen, and stored at –80°C for metabolomic and transcriptomic analysis. And in transcriptome analysis, a group of materials at moderate light (ML; 180 μmol/m2) for 12 h was added.

### Measuring total anthocyanin and flavonoid contents

Leaves from different treatment groups were weighed and pulverized. To measure total anthocyanins, each 0.1 g leaf tissue sample was combined with 0.1% hydrochloric acid methanol reagent and incubated at 40°C for 40 min to extract total anthocyanins. The optical densities at 530, 620, and 650 nm were measured with a UV spectrophotometer; 0.1 mol/L hydrochloric acid methanol solution was used as a blank control. Total anthocyanin content was calculated according to Greey’s formula (Lee and Ohnishi, 2003; Nitta et al., 2005b). To measure total flavonoids, perilla leaf powder was combined with an equal volume of 70% ethanol, and 1 mL of the extract and 1 mL of 5% sodium nitrite solution were added to a volumetric bottle. After shaking well for 6 min, 1 mL of 10% aluminum nitrate test solution was added to the flask, which was then mixed for 6 min. After adding 10 mL of sodium hydroxide test solution, the sample was mixed. Finally, 70% ethanol was added to bring the volume to 25 ml. After 15 min, the absorbance of the sample at 500 nm was measured by UV-vis spectrophotometry with 70% ethanol as a blank control. The rutin was used as the standard curve to calculate the content of total flavonoids.

### Metabolomics sample preparation and detection

The freeze-dried samples were crushed, and 100 mg of powdered tissue was combined with 1.2 mL of 70% methanol. After centrifuging at 12,000 rpm for 10 min, the extracts were filtered (SCAA-104, 0.22 μm pore size), and the metabolites in the extracts were analyzed used an UPLC-ESI-MS/MS system (UPLC, SHIMADZU Nexera X2; MS, Applied Biosystems 4500 Q TRAP). The conditions were as follows: UPLC: column, Agilent SB-C18 (1.8 μm, 2.1 mm * 100 mm). The mobile phase consisted of solvent A (pure water with 0.1% formic acid) and solvent B (acetonitrile with 0.1% formic acid). Sample measurements were performed with a gradient program. The column temperature was set to 40°C and the injection volume was 4 μL. The effluent was alternatively connected to an ESI-triple quadrupole-linear ion trap (QTRAP)-MS. LIT, and triple quadrupole (QQQ) scans were acquired on a triple quadrupole-linear ion trap mass spectrometer (Q TRAP), AB4500 Q TRAP UPLC/MS/MS System, equipped with an ESI Turbo Ion-Spray interface, operating in positive and negative ion mode, and controlled by Analyst 1.6.3 software (AB Sciex). The ESI source operation parameters were as follows: ion source, turbo spray; source temperature 550°C; ion spray voltage (IS) 5500 V (positive ion mode)/–4500 V (negative ion mode); ion source gas I (GSI), gas II (GSII), and curtain gas (CUR) were set at 50, 60, and 25.0 psi, respectively; the collision-activated dissociation (CAD) was high. Instrument tuning and mass calibration were performed with 10 and 100 μmol/L polypropylene glycol solution in QQQ and LIT mode, respectively. QQQ scans were acquired as multiple reaction monitoring (MRM) experiments with collision gas (nitrogen) set to moderate. Positive ion mode optimizes cluster removal voltage (DP) and Collision electric (CE) for individual MRM transitions were performed by further DP and CE optimization. A specific set of MRM transitions was monitored for each period based on the metabolites eluted within that period.

### RNA sequencing and annotation

A Tiangen RNA Prep Pure Kit for Plants was used to extract total RNA from *P. frutescens* leaves. A NanoDrop 2000 spectrophotometer (Thermo Scientific, Wilmington, DE, United States) was used to measure total RNA concentration and quality. RNA samples that met the requirements were sent to Wuhan Bena Technology Service Co., Ltd. (Wuhan, China) for transcriptomic sequencing. RNA-seq libraries (*P. frutescens* leaves collected at WL, ML12 h, ML6d, and RL × 3 replicates) were constructed and sequenced using the Illumina HiSeq4000 platform, and clean reads were mapped to the perilla reference genome using HISAT with default parameters.

### Identification of differential metabolites and differentially expressed genes

The metabolome data in this study was identified with chemical library MWDB (Metware Database), which were established by Metware Metabolism Company. Materials was quantitative based on secondary spectrum information, and isotope signals were removed during analysis, which contained K + ions and Na + ionsRepeated signals of NH4 + ions and fragments of other substances with larger molecular weight were identified. Metabolites with significantly different levels between groups were determined based on Variable Importance in Projection (VIP) ≥ 1 and absolute log2FC (fold change) ≥ 1. VIP values were extracted from the OPLS-DA results, and score plots and permutation plots were generated using the R package MetaboAnalystR. The data were log transformed (log2) and subjected to mean centering before OPLS-DA. To avoid overfitting, a permutation test (200 permutations) was performed. Gene expression levels were calculated as fragments per kilobase of transcript per million mapped reads (FPKM). Differentially expressed genes (DEGs) were identified from normalized read count data using DESeq2.34. Genes with | log2 (fold change) | ≥ 1 and *p* < 0.01 were considered to be DEGs.

### Hierarchical cluster analysis, Pearson correlation coefficients, and principal component analysis

The HCA (hierarchical cluster analysis) results of samples and metabolites were presented as heatmaps with dendrograms, while PCC (Pearson correlation coefficients) between samples were calculated by the core function in R and were presented only as heatmaps. Both HCA and PCC were carried out with the R package pheatmap. For HCA, normalized signal intensities of metabolites (unit variance scaling) were visualized as a color spectrum. Unsupervised PCA (principal component analysis) was performed using the statistics function prcomp within R^1^. The data were unit variance scaled prior to unsupervised PCA.

### Gene ontology enrichment and Kyoto encyclopedia of genes and genomes enrichment analysis

The identified metabolites were annotated using the Kyoto Encyclopedia of Genes and Genomes (KEGG) Compound database^2^. Gene function annotation was performed using four databases: National Center for Biotechnology Information (NCBI^3^), non-redundant protein sequences (NR^4^), the Swiss-Prot protein sequence database, and Gene Ontology (GO^5^). The annotated metabolites and genes were then mapped to the KEGG Pathway database^6^. Pathways with significantly regulated metabolites were subjected to MSEA (metabolite sets enrichment analysis), and their significance was determined based on *p*-values from hypergeometric tests. Finally, the DEGs in the WL, ML12h, ML6d, and RL groups were subjected to GO and KEGG pathway enrichment analysis.

### Weighted gene co-expression network analysis of metabolome and transcriptomic data

The weighted gene co-expression network analysis (WGCNA) package was used to generate co-expression network modules between metabolites and genes. Using the automatic network construction function (blockwise Modules) with default parameters, co-expression modules were obtained based on TOM (topological overlap measure). The initial clusters were merged on eigengenes. The eigengene value was calculated for each module and used to search for associations with key anthocyanin substances. The transcriptional regulatory networks were generated by combining the Pearson correlation coefficient (PCC > 0.85) between genes and transcription factors, and the *cis*-element binding sites were predicted in the promoter regions of key anthocyanin genes in the same module. The networks were visualized by CYTOSCAPE (v.3.7.2, United States) (Kohl et al., 2011).

### Phylogenetic analysis

The conservative domain of the candidate MYBs and bZIPs from perilla and other plants were used to construct phylogenetic. Accession numbers of MYBs and bZIPs from other plants are shown in Supplementary Table S8. The neighbor-joining (NJ) tree was constructed using the poison model with MEGA-X with 1000 bootstrap replicates. The phylogenetic trees were visualized using the iTOL web tool^7^.

### Quantitative reverse-transcription PCR

Quantitative reverse-transcription PCR was performed as previously described (Livak and Schmittgen, 2001). Total RNA was isolated from the samples using a MAGEN RNA Extraction Kit (MAGEN, United States), and reverse transcription was performed using an Evo M-WLV RT Kit (Accurate, Hunan, China). Primers for each gene (*ANS*, *CHI*, *DFR*, and so on) were designed using Primer 5.0; the primers are listed in Supplementary Table S9. The specificity of the primers was verified by agarose gel electrophoresis. PCR amplification was performed using a LightCycler 480 II REAL-TIME PCR system (Roche, Basel, Switzerland). The qPCR cycling conditions were 1 cycle of pre-denaturation at 95°C, 30 s; 40 cycles of 95°C for 5 s and 60°C for 30 s; dissolution at 95°C for 5 s; 60°C for 1 min. All genes were amplified with three replicates, and the perilla *Actin* gene was used as an internal reference gene.

## Results

### Various anthocyanin content of perilla under different light intensity treatments

**FIGURE 1:**
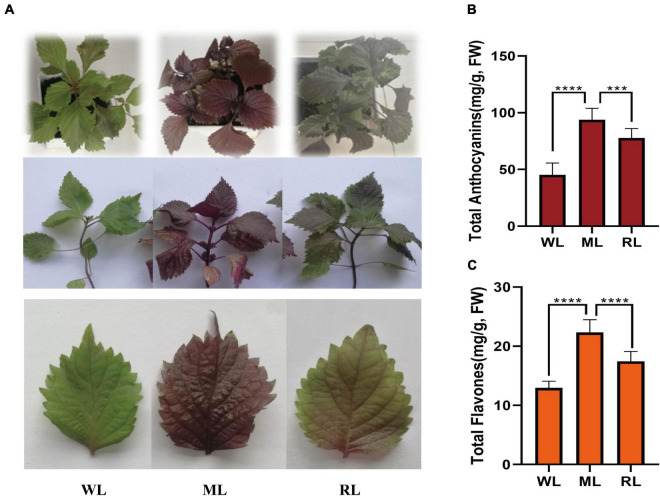
Leaf color, anthocyanin, and flavone contents of perilla under different light intensity treatments. **(A)** Phenotypes of perilla. **(B)** Total anthocyanin contents of perilla leaves. **(C)** Total flavone contents of perilla leaves under WL, weak light; ML, moderate light for 6 days; RL, recovered weak light for 6 days. **** Indicates *P* value < 0.0001.

To identify the leaves color and major pigment components under different light, we observed and measured the total anthocyanin and total flavonoid contents during different light intensity treatments. The perilla leaves color under different light intensities were obviously affected. The leaves and seedlings of the light-sensitive red perilla variety were green when cultured under weak light intensity (15 μmol/m^2^, WL). After transferring to moderate light intensity (180 μmol/m^2^, ML) for 6 days, the leaves and whole plants turned dark red. When returned to weak light intensity for 6 days (15 μmol/m^2^, RL), the leaf color gradually returned to partially green (Figure 1A). Like the changes of the leaf color, total anthocyanin and total flavone levels significantly increased under ML treatment and then decreased in RL compared with WL (Figures 1B,C). These results demonstrate that light intensity significantly affects the leaf color and the accumulation of total anthocyanins and total flavones in leaves of the light-sensitive perilla variety.

### Metabolome detection under different light intensity treatments

**FIGURE 2:**
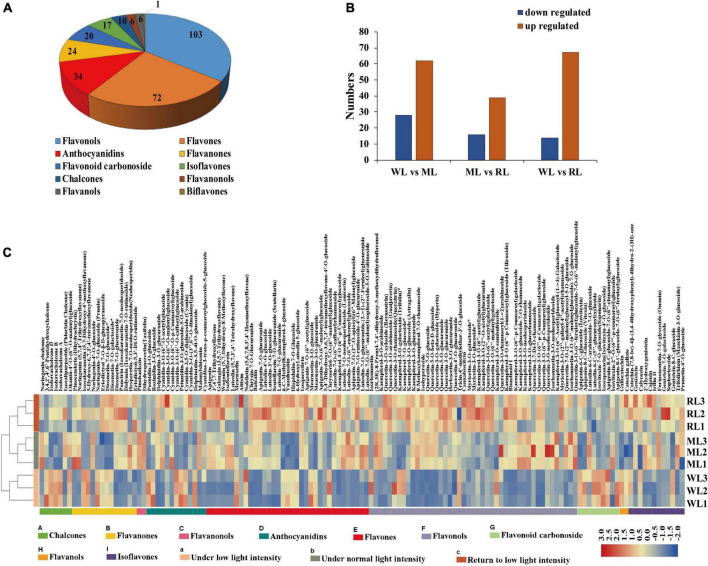
The flavonoid metabolites of perilla leaves under different light intensity treatments. **(A)** Types of flavonoid metabolites detected. **(B)** Differentially expressed metabolites (DEMs) in WL vs. ML6d, ML 6d vs. RL, and WL vs. RL. **(C)** Contents of flavonoid metabolites in perilla leaves under different light intensity treatments.

To assess anthocyanin components under different light treatments, we performed metabolomic analysis using UPLC-ESI-MS/MS. The samples were clearly clustered by PCA (Supplementary Figure S1). In total, 293 flavonoid metabolites were identified, including 103 flavonols, 72 flavones, 34 anthocyanins, and 84 other derivatives (Figure 2A; Supplementary Table S1). There were 90, 55, and 81 differentially expressed metabolites (DEM) in WL vs. ML, ML vs. RL, and WL vs. RL, respectively (Figure 2B).

Quantitative analysis showed that approximately 140 (48%) flavonoid metabolites were affected under different light intensity treatments in perilla leaves. Among these, 62 metabolites were significantly induced under increased light intensity, including 23 flavonols, 19 flavones, and 5 anthocyanins (Supplementary Table S2). Among them, the contents of malonylshisonin increased 1.7-fold under ML treatment compared with WL and decreased 0.8-fold under RL treatment compared with ML (Supplementary Table S3). Naringin chalcone and dihydroquercetin (early intermediates in the anthocyanin biosynthesis pathway), as well as cyanidin-3,5-*O*-diglucoside, cyanidin-3-*O*-(6”-*O*-*p*-coumaryl) glucoside-5-*O*-glucoside, and cyanidin-3-*O*-(6”-*O*-*p*-coumaryl) glucoside (important precursors of malonylshisonin) were all detected in this study. Interestingly, the levels of naringin chalcone, dihydroquercetin, and cyanidin-3-*O*-(6”-*O*-*p*-coumaryl) glucoside increased by 4.3-, 5.8-, and 3.2-fold (Supplementary Table S3), respectively, under ML treatment compared with WL. By contrast, the cyanidin-3,5-*O*-diglucoside contents decreased by 0.6-fold under ML treatment compared with WL. These results suggest that light intensity affects accumulation of the metabolites in anthocyanin biosynthesis pathway in perilla.

According to the change trends of flavonoid metabolites under different treatments, they were divided into eight categories. The levels of subclass 4 and subclass 7 members initially increased, then decreased, possessed consistent pattern with total anthocyanin contents. Both malonylshisonin and naringin chalcone belong to subclass 7. The levels of subclass 2 and subclass 6 members continuously increased under ML and RL treatment (Supplementary Figure S2). The cyanidin-3-*O*-(6”-*O-P*-coumaryl) glucoside-5-*O*-glucoside and dihydroquercetin belong to subclass 2 and subclass 4, respectively. The four subclasses attracted our main attention.

### Transcriptomic analysis under different light intensity treatments

To explore the molecular regulatory mechanism of the perilla anthocyanin metabolites in response to light intensity, we performed transcriptomic sequence. Considering the rapid response of transcriptomic level to light treatment, we added a short treatment (12 h) under 180 μmol/m^2^ of light (ML12h). After RNA sequencing and data filtering, 701.64 million clean reads with 105 Gb clean data were obtained. The Q20 reached 97%, with an average GC content of 48%. When we mapped the sequencing reads to perilla reference genomes, we obtained an average unique mapping ratio of 82.94% and a multiple mapping ratio of 10.19% (Supplementary Table S4).

**FIGURE 3:**
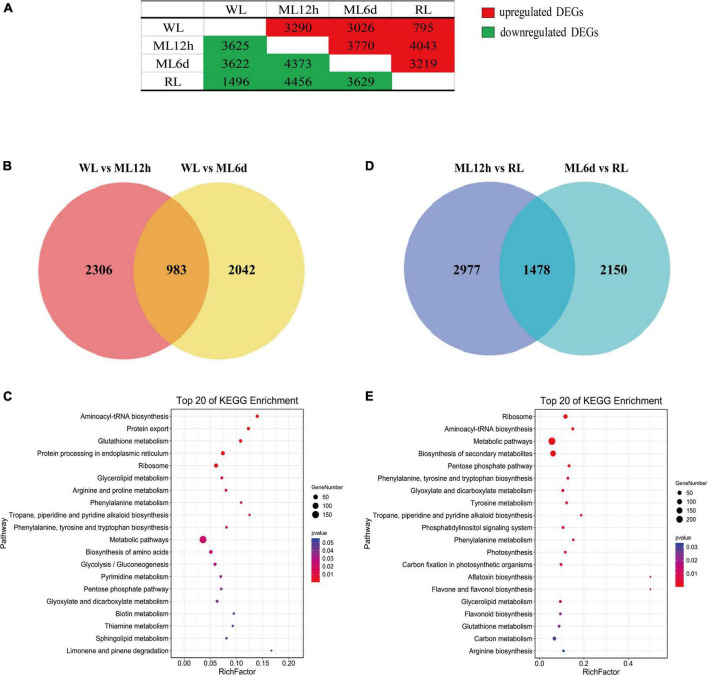
Venn diagram and KEGG pathways of the DEGs under different light intensity treatments. **(A)** Number of upregulated and downregulated differentially expressed genes (DEGs) in pair-wise comparisons. **(B)** Venn diagram and **(C)** top 20 KEGG pathways of upregulated genes of the ML12h and ML6d groups compared with WL. **(D)** Venn diagram and **(E)** top 20 KEGG pathways of downregulated genes in the RL group compared with ML12h and ML6d.

Subsequently, we identified DEGs among all samples. And most of DEGs were responsed to light intensity showing to the results of GO analysis (Supplementary Figure S3). Compared to the WL group, 3290 and 3026 genes were upregulated in ML12h and ML6d, respectively. Compared to the RL group, 4043 and 3219 genes were upregulated in ML12h and ML6d, respectively (Figure 3A). Among the upregulated genes under ML treatment, 983 genes showed increased expression under short (12 h) and long (6 days) treatments compared with WL (Figure 3B). These DEGs are mainly involved in metabolic pathways, phenylalanine and tyrosine tryptophan biosynthesis and phenylalanine metabolism in the KEGG enrichment (Figure 3C). By contrast, 1478 genes were downregulated in RL compared with ML (Figure 3D). The down-regulated genes were enriched in biosynthesis of secondary metabolites, phenylalanine and tyrosine tryptophan biosynthesis, tyrosine metabolism, phenylalanine metabolism, and flavonoid biosynthesis in the KEGG enrichments (Figure 3E). Based on the expression pattern, we divided all DEGs into 26 categories (Supplementary Figure S4). The 983 and 1478 DEGs most belong to Profile 20 (upregulated in ML12h and ML6d compared with WL) and Profile 23 (downregulated in RL compared with ML12h and ML6d), respectively. These photo-response genes attracted our main attention.

### The metabolites and key genes in the anthocyanin biosynthesis pathway

**FIGURE 4:**
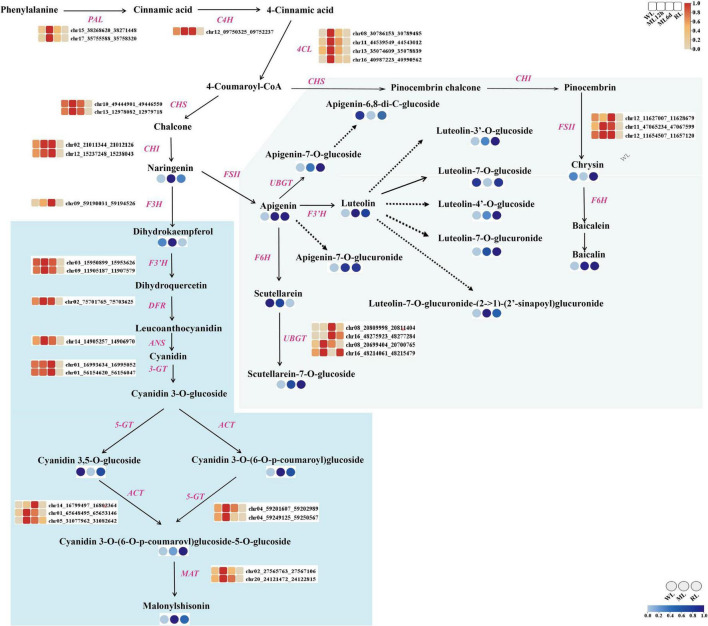
The metabolites and genes involving in the anthocyanin biosynthesis pathway under different light intensity treatments in perilla leaves. The blue background indicates the major malonylshisonin biosynthetic pathway. The green background indicates the major flavonoid biosynthetic pathway. The blue color in the circle indicates the level of the metabolite under WL, ML, and RL treatment. The red color in the blocks represents the relative expression levels of genes under WL, ML12h, ML6d, and RL treatments. PAL, phenylalanine ammonia lyase; C4H, cinnamate 4-hydroxylase; 4CL, 4-coumarate-CoA ligase; CHS, chalcone synthase; CHI, chalcone isomerase; F3H, flavanone 3-hydroxylase; F3’H, flavonoid 3’-hydroxylase; DFR, dihydroflavonol reductase; ANS, anthocyanin synthase (leucoanthocyanin dioxgenase); 3-GT, flavonoid 3-glucosyltransferase; ACT, anthocyanin acyltransferase; 5-GT, anthocyanin 5-glucosyltransferase; MAT, anthocyanin malonyltransferase.

We identified metabolites and genes involved in the anthocyanin and flavones biosynthetic pathways (Figure 4). Malonylshisonin biosynthesis involves 10 catalytic reactions, containing the initial cyanidin biosynthesis and the latter cyanidin derivatization pathway (Saito and Yamazaki, 2002; Jiang et al., 2020). Naringin chalcone, dihydroquercetin, cyanidin-3,5-*O*-diglucoside, cyanidin-3-*O*-(6”-*O*-*p*-Coumaroyl) glucoside, cyanidin-3-*O*-(6”-*O*-*p*-Coumaroyl) glucoside-5-*O*-diglucoside, and malonylshisonin were identified in the metabolomic analysis. Except for cyanidin 3,5-*O*-glucoside, these metabolites obviously accumulated under increased light intensity. Then, we identified 95 genes encoding the 10 anthocyanin synthases in the malonyshisonin biosynthetic pathway (Figure 4 and Supplementary Table S5). Furthermore, 32 genes were upregulated after light treatment, which encode the key rate-limiting enzymes in the basic anthocyanin biosynthetic pathway. Among these, 18 genes (including *PAL, 4CL, CHS, F3’H, ANS, 5-GT, and MAT*) were more strongly upregulated under 12 h ML treatment, and five genes (including *F3H, 3-GT, ACT, and CHI*) had higher expression levels under 6 days ML treatment. Only one *C4H* gene was expressed at high levels under both 12 h and 6 days ML treatments. These results suggest that light intensity remarkably influences the gene expression and metabolite accumulation involving in anthocyanin biosynthesis in perilla leaves.

Moreover, we identified 14 light-regulated flavones based on analysis of the flavonoid biosynthesis pathway. Most of these compounds accumulated under light treatment. Specifically, the contents of luteolin, apigenin, and baicalin increased by 5.0-, 5.4-, and 11.6-fold, respectively, under ML treatment compared with WL. Comparatively, the levels of apigenin-6,8-di-C-glucoside, luteolin-7-*O*-glucoside, and chrysin decreased under ML treatment. Because many genes involved in the accumulation of these substances are unknown, we only detected *FSII*, *F6H*, and *UBGT* in the flavone biosynthetic pathway (Supplementary Table S5). Like the malonylshisonin biosynthesis genes, both *FSII* and *UBGT* were upregulated under ML treatment compared with WL. In view of luteolin, apigenin and more flavones possess pharmacological effects (Lei et al., 2020; Liang et al., 2020; Rizzo et al., 2021), the results show that strongly accumulated flavones under elevated light treatment, with provided potential application for further research.

### Integrated transcriptomic and metabolomic analysis

**FIGURE 5:**
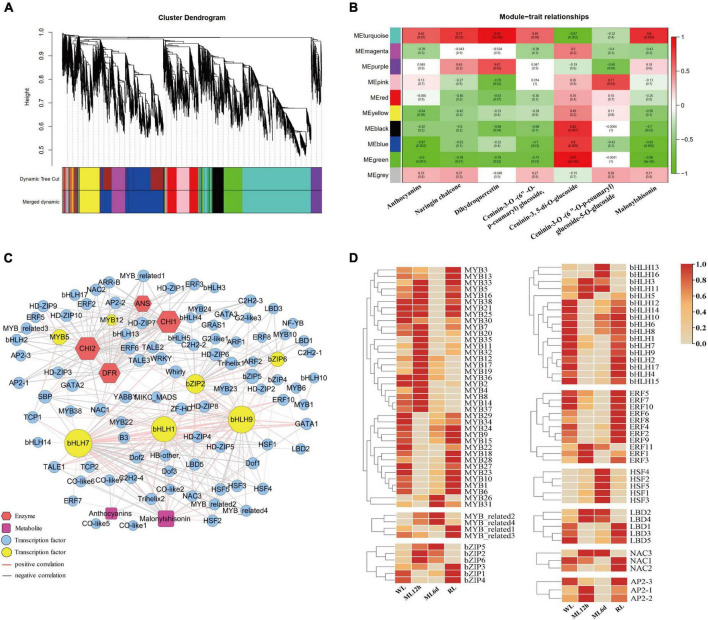
Weighted correlation network analysis (WGCNA) of the anthocyanin biosynthetic pathway. **(A)** Dendrogram of the co-expression modules (clusters). The major tree branches constitute 10 modules labeled with different colors. **(B)** Heat map showing the correlations between modules and anthocyanin compounds. Red indicates a positive correlation, and green indicates a negative correlation between the cluster and the sample. **(C)** Co-expression network of the genes and metabolites with correlation coefficients greater than 0.85. **(D)** Heat maps of the expression levels of genes encoding MYBs, MYB-related, bZIPs, bHLHs, ERFs, HSFs, LBDs, NACs, and AP2s based on the transcriptomic data.

To gain further insight into the regulation of anthocyanin biosynthesis in response to light intensity, we performed WGCNA to identify co-expression networks between anthocyanin metabolites and gene expression levels. Ten co-expression modules were identified based on their similar expression patterns (Figure 5A). The turquoise module is positively correlated with anthocyanins, malonylshisonin, and other metabolites (Figure 5B). We therefore focused on those genes in the turquoise module. We identified anthocyanin biosynthesis and transcription factor genes in this module and used gene pairs with correlation coefficients more than 0.85 to construct a regulatory network (Figure 5C and Supplementary Table S7). Five important genes involved in anthocyanin biosynthesis were identified in the turquoise module, including *ANS*, *4CL*, *DFR*, and *CHI*, which were positively correlated with total anthocyanin and malonylshisonin contents. The 147 transcription factors were also identified, mainly encoding MYB, bHLH, bZIP, ERF, and NAC in the module. Notably, *MYB5*, *MYB12*, *bZIP2*, *bZIP6, bHLH1*, *bHLH7*, and *bHLH9* had high connectivity with malonylshisonin and five anthocyanin biosynthesis genes. The MYB_related2, MYB_related4 had high positive correlation with malonylshisonin. Moreover, the expression level of most identified MYB and bZIP genes were strongly upregulated under ML treatment compared with WL and downregulated under RL treatment compared with ML (Figure 5D). By contrast, *MYB22, bHLH1*, *bHLH7*, and *bHLH9* were negatively correlated with malonylshisonin content. *MYB22* and *bHLH1* were also negatively correlated with total anthocyanin contents (Supplementary Table S6). Hence, these transcription factors were speculated to play important roles in regulating malonylshisonin accumulation under different light intensities.

### Candidate MYB genes involved in anthocyanin biosynthesis

MYB transcription factors are major members of MBW ternary transcriptional complex, which is an important regulator of anthocyanin metabolism in many plants (Lloyd et al., 2017; Li et al., 2020; Wang et al., 2020; Zhang et al., 2020; Ning et al., 2021). In WGCNA analysis, we identified 38 MYB transcription factors, containing 37 R2R3-MYB subfamily members and one 1R-MYB (MYB10). These MYBs showed high correlation coefficient with malonylshisonin and five anthocyanin biosynthesis genes (Supplementary Figure S5). Among them, 22 R2R3-MYB members were highly induced under ML treatment, while 15 were downregulated in response to light treatment (Figure 5D).

**FIGURE 6:**
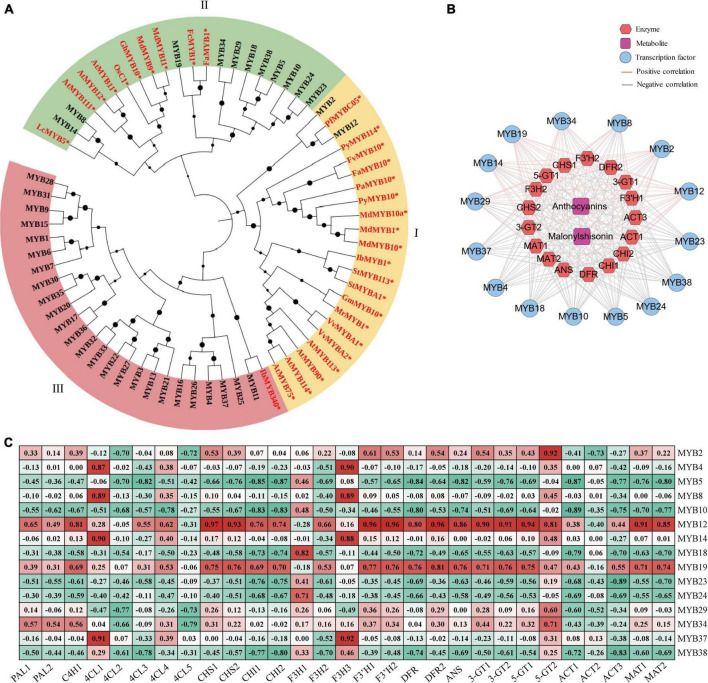
The MYB transcription factors involved in anthocyanin biosynthesis in perilla. **(A)** Phylogenetic relationships of MYBs between perilla and other plants. Yellow, green, and pink background indicate three subclasses in the evolutionary tree. The dots next to the nodes represent bootstrap values of 1000 replicates. The red font and symbol * indicate that these objects have been reported. **(B)** Co-expression networks between candidate MYBs. **(C)** Correlation coefficients between candidate MYB transcription factors and key anthocyanin biosynthesis genes. At, *Arabidopsis thaliana*; Vv, *Vitis vinifera*; St, *Solanum tuberosum*; Pb, *Pyrus* x *bretschneideri*; Os, *Oryza sativa* Japonica; Pf, *Perilla frutescens* var. *crispa*; Pa, *Prunus avium*; Py, *Pyrus pyrifolia* var. *culta*; Mr, *Morella rubra*; Md, *Malus domestica*; Lc, *Litchi chinensis*; Ib, *Ipomoea batatas*; Gh, *Gossypium hirsutum*; Gm, *Garcinia mangostana*; Fa, *Fragaria* x *ananassa*; Fv, *Fragaria vesca*; Fc, *Fragaria chiloensis*.

To examine the MYB transcription factors involved in regulating anthocyanin accumulation, we constructed a phylogenetic tree combined the MYBs that we identified in perilla and were previously reported to regulate anthocyanin accumulation in other plants. The phylogenetic tree was divided into three subclasses (Figure 6A). Subclass I contains perilla MYB12, MYB2 and key anthocyanin-regulated MYBs from Arabidopsis and apple. Subclass II includes 11 perilla MYBs and three Arabidopsis R2R3-MYB family members. Among them, *MYB8* and *MYB14* showed peak expression under ML12h treatment. Subclass III includes 25 MYBs from perilla and IbMYB340. Among them, *MYB4* and *MYB37* were expressed at high levels and responded to light treatment.

For further explore the relationships between the MYBs and anthocyanin biosynthesis, we performed correlation analysis of 15 selected MYBs with anthocyanin metabolites and 17 key anthocyanin biosynthesis genes. Among these, 9 MYBs showed positive correlations with anthocyanin metabolites and 6 showed positive correlations with key anthocyanin biosynthesis genes (Figure 6B). Interestingly, *MYB12* and *MYB19* showed significantly positive correlations with most anthocyanin biosynthesis genes (Figure 6C). The *MYB12* had been reported in malonylshisonin formation, which was found in responsive to light treatment in the study.

Furthermore, the *cis*-acting elements of four key anthocyanin biosynthesis genes, including *DFR*, *CHI1*, *CHI2*, and *ANS*, were analyzed. Several light-response elements and hormone-response elements were identified in the four anthocyanin genes (Supplementary Figure S7). Interestingly, we identified CCAAT-box and MYB-recognition element (MRE) *cis*-acting elements in the promoter regions of perilla *CHI1* and *ANS*, respectively (Supplementary Figure S7). Combining WGCNA analysis, phylogenetic analysis, and *cis*-acting element analysis, more MYB transcription factors were predicted to regulate malonylshisonin accumulation under the light treatment in perilla.

### Candidate bZIP genes involved in anthocyanin biosynthesis

**FIGURE 7:**
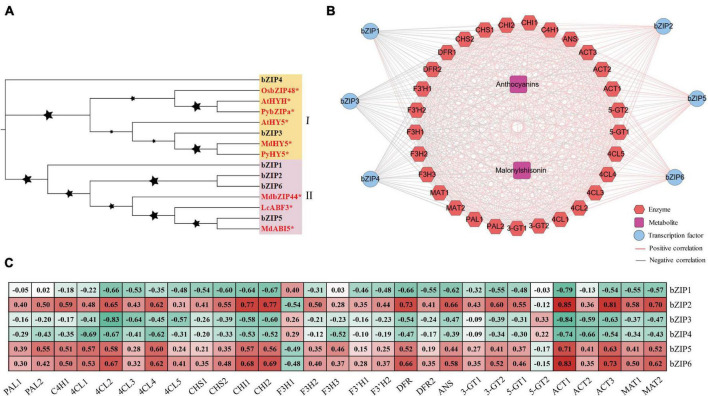
The bZIP transcription factors involved in the light response and anthocyanin regulation in perilla. **(A)** Phylogenetic relationships of bZIP transcription factors. Yellow and pink background indicate two subclasses in the evolutionary tree. The stars next to the nodes represent bootstrap values of 1000 replicates. The red font and symbol * indicate that these objects have been reported. **(B)** Co-expression networks between candidate bZIPs. **(C)** Correlation coefficients between candidate bZIP transcription factors and key anthocyanin biosynthesis genes. At, *Arabidopsis thaliana*; Py, *Pyrus pyrifolia*; Os, *Oryza sativa* Japonica; Md, *Malus domestica*; Lc, *Litchi chinensis*.

The bZIP transcription factors were recently reported to regulate anthocyanin biosynthesis (Hu et al., 2019; Liu et al., 2019; Wang et al., 2020). In the turquoise module, we also identified 6 bZIP transcription factors (Supplementary Figure S6). These perilla bZIPs were divided into two subclasses in the evolutionary tree (Figure 7A). Subclass I consist of bZIP4 and bZIP3 from perilla, along with HY5 transcription factors from other plants. Subclass II consists of 4 bZIP transcription factors from perilla, along with MdbZIP44, LcABF3, and MdABI5. Co-expression analysis showed that *bZIP2*, *bZIP5*, and *bZIP6* share positive regulatory relationships with key anthocyanin biosynthesis genes (*CHI1*, *CHI2*, *ANS*, and *DFR*), while *bZIP1*, *bZIP3*, and *bZIP4* share negative regulatory relationships with these genes (Figure 7B). Interestingly, *bZIP2*, *bZIP5*, and *bZIP6* showed significant positive correlations with total anthocyanin and malonylshisonin contents and most anthocyanin biosynthesis genes (Figure 7C). The promoter sequences of *CHI1*, *CHI2*, *ANS*, and *DFR* all contain G-boxes, a light-responsive binding site that is recognized by bZIP transcription factors (Supplementary Figure S7). The *bZIP2* and *bZIP6* were identified to encode G-box binding factor 3 in perilla, suggesting that they may play key roles in the light response and the anthocyanin regulation.

### Quantitative RT-PCR analysis of the candidate genes involved in anthocyanin biosynthesis in *Perilla frutescens*

**FIGURE 8:**
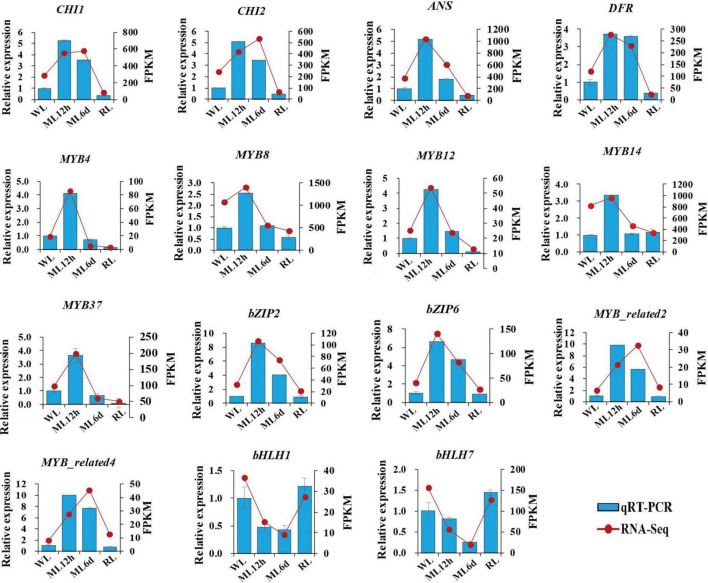
Relative expression levels of candidate genes. The histogram indicates gene expression data form qRT-PCR. The line chart indicates FPKM from transcriptomic sequence. Data are presented as means ± standard deviation (*n* = 3).

We measured the expression levels of anthocyanin biosynthesis genes (*CHI1*, *CHI2*, *ANS*, and *DFR*) and important transcription factor genes (*MYB*, *bZIP*, and *bHLH*) involved in anthocyanin regulation by qRT-PCR (Figure 8). Combined the transcriptomic and qRT-PCR data, the candidate genes showed similar expression patterns. The anthocyanin biosynthesis genes (*CHI1*, *CHI2*, *ANS*, and *DFR*), MYB genes, and bZIP genes were upregulated under increased light intensity. These genes were all upregulated after a short (12 h) moderate light intensity treatment and downregulated after a long treatment (6 days), and their expression continued to decline under low light intensity. The response of the anthocyanin biosynthesis genes to light intensity treatment might be the direct cause of accumulation of anthocyanin metabolites. The MYB transcription factor genes were more sensitive to short vs. long light treatment, pointing to their important regulatory functions in anthocyanin metabolite accumulation. The *bZIP2* and *bZIP6* are homologous to the important light-response transcription factor gene *HY5*. Both were upregulated after a short light treatment. We also identified *bHLH1* and *bHLH7* transcription factor genes in perilla. Interestingly, both genes were downregulated under light treatment. The *bHLH1* and *bHLH7* encode PIF3 and PIF7, respectively. The PIFs were negatively regulation factors in response to light in other plants. The downregulated trend of *bHLH1* and *bHLH7* under light treatment is consistent with their functions. The expression patterns of key anthocyanin biosynthesis genes and transcription factors in response to light points to their important roles in regulating anthocyanin biosynthesis.

## Discussion

Leaf color and anthocyanin accumulation are important traits for the medicinal and ornamental value of perilla (Ishikura, 1981; Yu et al., 2017). Perilla leaves color is highly sensitive to light conditions, providing an opportunity to study the molecule mechanism of anthocyanin biosynthesis and regulation (Mastropasqua et al., 2020).

In this study, we performed metabolomic and transcriptomic analyses of the light-sensitive perilla variety under different light intensity treatments. The metabolomic analysis identified 293 flavonoid compounds. Focus on malonylshisonin, the major anthocyanin metabolites in perilla, obviously increased in response to increased light treatment. Malonylshisonin biosynthesis involves 10 catalytic reactions, containing the initial cyanidin biosynthesis and the latter cyanidin derivatization pathway. In the latter steps, cyanidin-3-*O*-glucoside is converted to cyanidin-3-*O*-(6”-*O*-*p*-coumaryl) glucoside and cyanidin 3,5-*O*-glucoside (Springob et al., 2003). Under ML treatment, the content of cyanidin 3,5-*O*-glucoside was decreased, whereas the content of cyanidin-3-*O*-(6”-*O*-*P*-coumaryl) glucoside was increased, suggesting that the metabolic flow favored the formation of cyanidin-3-*O*-(6”-*O*-*P*-coumaryl) glucoside under light treatment. Next, we investigated the biosynthesis genes of malonylshisonin accumulation under different light treatments. Previous work found that nearly all the genes encoding the enzymes (except for *CHI*) showed higher expression in red perilla than in green perilla (Jiang et al., 2020). In the current study, the genes encoding CHS, CHI, F3H, DFR, and ANS, the rate-limiting enzymes in the anthocyanin biosynthetic pathway (Takos et al., 2006), were obviously responded to light treatment. These genes may play important roles in malonylshisonin accumulation in perilla leaves under light treatment.

In the WGCNA and correlation analysis, *ANS*, *CHI*, and *DFR* were positively correlated with malonylshisonin content. The homologous proteins of ANS, CHI, and DFR were responded to light treatment in other plants (Britsch, 1990; Menssen et al., 1990; Saito et al., 1999). CHI is the rate-limiting enzyme for flavonoid biosynthesis (Bednar and Hadcock, 1988; McKhann et al., 1998; Shimada et al., 2003). The expression of *CHI* increases in rice in response to high light levels and UV irradiation (Albert et al., 2009; Park et al., 2021). DFR catalyzes the conversion of dihydroquercetin to leucoanthocyanidin, which is recognized as a pivotal step in anthocyanin biosynthesis (Xie et al., 2004; Miyagawa et al., 2015; Ni et al., 2020). The expression of *DFR* is downregulated in the shade in *Brassica oleracea* (Liu et al., 2020). ANS catalyzes the oxidation of leucoanthocyanidin (Turnbull et al., 2000, 2003), and *ANS* is upregulated by high-light treatment in *Arabidopsis thaliana* (Zheng et al., 2019, 2021). In the current study, the expression levels of the perilla genes *CHI1*, *CHI2*, *DFR*, and *ANS* all increased under ML treatment. These results indicate that these genes play a major role in determining malonylshisonin biosynthesis in different light intensities.

Plants respond to light via photoreceptors and employ multiple transcription factors to regulate anthocyanin metabolites formation (Zhang et al., 2021). To further identify the key regulation factors, we used WGCNA method to comprehensively analyze metabolites and key genes in perilla. In this study, the 147 transcription factors were also identified based on WGCNA analysis. Among them, R2R3-MYBs have been widely researched. There are four Arabidopsis thaliana R2R3-MYB family members (AtMYB113, AtMYB90, AtMYB114, and AtMYB75) were reported control anthocyanin biosynthesis in vegetative tissues (Gonzalez et al., 2008). AtMYB75 (also called PAP1) activates the transcription of *CHS*, *DFR*, and *ANS* (Shi and Xie, 2010, 2011) and participates in the regulation of anthocyanin biosynthesis by HY5 via the transcriptional activation of its promoter (Shin et al., 2013). The MdMYB10 and MdMYB1 are key anthocyanin-related transcription factors in apple (Takos et al., 2006; Espley et al., 2007). In apple, the expression of *MdMYB1* and its allele *MdMYB10* is induced by sunlight (Takos et al., 2006; Feng et al., 2013). The expression of *MdMYB10* is also regulated by HY5, which binds to the G-box in the *MdMYB10* promoter in a light-responsive manner, thereby affecting the expression of downstream structural genes and anthocyanin accumulation in apple (An et al., 2017). Interestingly, PfMYB12 were identified shares complete sequence identity with MYBC05. The MYBC05 was previously reported to promote the production of anthocyanins, which is thought to bind to myC-F3G1 and PFWD to form the MBW complex, which promotes the production of anthocyanins in red perilla (Yamazaki et al., 2003; Yamazaki and Saito, 2011). Hence, the PfMYB12 and PfMYB2 are clustered with above MYBs, which are thought to important function in anthocyanin light-responsive accumulation. Next, The PfMYB8 and PfMYB14 were clustered with three AtMYBs (AtMYB11, AtMYB12, AtMYB111), which targeted several flavonoid biosynthesis genes, including *CHS*, *CHI*, *F3H*, and *FLS1* in Arabidopsis (Stracke et al., 2007). AtMYB12 and AtMYB111 also control flavonol accumulation in roots in a light-dependent manner (Stracke et al., 2017). PfMYB4 and PfMYB37 were clustered with IbMYB340, which interacted with IbERF71 and IbbHLH2 to regulate anthocyanin biosynthesis in sweet potato (Ning et al., 2021). Moreover, MYB-binding sites, including MBS, MRE, and CCAAT-box elements, were identified in the promotors of *CHI1*, *CHI2*, and *ANS*. The CCAAT-box is the binding site of the HvMYB1 transcription factor in barley (Alexander et al., 2019). The MRE cis-acting elements is recognized and bound by BrPAP1 transcription factor, which regulates anthocyanin biosynthesis in *Brassica rapa* (Yang et al., 2021). These findings suggest that these MYBs play important roles in regulating anthocyanin accumulation in perilla in response to different light intensities. To sum up, the six PfMYB (PfMYB2, PfMYB12, PfMYB8, PfMYB14, PfMYB4, and PfMYB37) were predicted to possess important function in photosensitive anthocyanin regulation in perilla.

The bZIP family, one of the largest transcription factor families in plants, is involved in physiological processes such as plant development, environmental signaling, and stress responses (Djamei et al., 2007; Liu C. C. et al., 2018; Xiao et al., 2021). The bZIP transcription factor HY5 is an important transcription factor involved in light signal transduction and plant pigment accumulation in response to light (Holm et al., 2002; Stracke et al., 2010; Shin et al., 2013; Gangappa and Botto, 2016). HY5 also promotes flavonoid accumulation in response to both visible and UV-B light. Using WGCNA, we identified six bZIP transcription factors in the co-expression modules. Perilla bZIP3 and bZIP4 clustered with HY5s from other plants. In the previous research, HY5 could responds to receptors for blue, red/far-red, and ultraviolet light (Oyama et al., 1997; Ang et al., 1998; Chattopadhyay et al., 1998; Brown and Jenkins, 2008) and directly binds to G-box or ACE-box elements in the promoters of MYB genes to promote their expression to promote flavonoid biosynthesis (Holm et al., 2002; Stracke et al., 2010; Shin et al., 2013). Perilla *bZIP3* and *bZIP4* were both induced by light but showed negative regulatory relationships with anthocyanin pathway genes in the current study. By contrast, *bZIP2* and *bZIP6* showed positive regulatory relationships with most anthocyanin biosynthesis genes and possess light-regulated traits in perilla. *bZIP2* and *bZIP6* encode G-box binding protein 3 (GBF3) transcription factors. In Arabidopsis, GBF3 has the same gene expression pattern as HY5, and its binding sites showed a 86.7% overlap with those of HY5 under changing light conditions, suggesting that GBF3 might interfere with the function of HY5 (Kurihara et al., 2020). bZIP2 and bZIP6 also clustered with MdbZIP44 and LcABF3. MdbZIP44 promotes anthocyanin accumulation in response to abscisic acid by enhancing the binding of MdMYB1 to the promoters of downstream target genes in apple (An et al., 2018). LcABF3 activates the promoter region of *LcMYB1* and structural genes such as *LcF3’H* and *LcDFR* to modulate anthocyanin biosynthesis in lychee (Hu et al., 2019). Interestingly, *bZIP2* and *bZIP6* had the highest correlations with *CHI1* and *CHI2* in WGCNA. The *CHI1* and *CHI2* promoters contain G-box *cis*-acting elements, which can be recognized and combined by bZIPs. That means bZIP2 and bZIP6 perhaps together with MYBs, activated the transcription of the key candidate anthocyanin biosynthesis genes and promote the accumulation of malonylshisonin under moderate light intensity. Moreover, the *bHLH1* and *bHLH7* encode PIF3 and PIF7. Phytochrome-interacting factor (PIF) genes are downregulated in the presence of light. These bHLH transcription factors function as primary partners of the red/far-red light receptors phytochromes in light signaling (Nozue et al., 2007). Our results indicate that bHLH1 and bHLH7 negatively regulate anthocyanin biosynthesis in the light, which is consistent with the function of PIFs in the light response.

In the research, we also identified 14 important flavone metabolites in perilla leaves, including luteolin, apigenin, baicalein, scutellarein, and their glycoside derivatives. Luteolin can attenuate allergic nasal inflammation (Jeon et al., 2014; Liang et al., 2020). Apigenin and apigenin-7-diglucuronide can mitigate oxidative stress and possesses anti-inflammatory activity (Bian et al., 2017; Kasiri et al., 2018). Baicalin represses C/EBP beta via redox homeostasis, representing a potentially effective treatment for Parkinson’s disease (Lei et al., 2020). They are all possess a certain of pharmacological functions and got more attention. The accumulation of 10 flavones increased in perilla leaves in response to moderate light treatment. This increase in the levels of pharmacologically important flavones indicates that the value of perilla can be increased under the proper light conditions. The flavonoid biosynthesis and its regulation require further study in perilla.

In summary, the regulation of light intensity signal in plants is a complex process. We mainly focused on the red perilla malonylshisonin synthesis pathway. In addition, the function of each candidate gene needs to be confirmed by different methods. Therefore, further work is required to elucidate the mechanism of light signal in perilla plants.

## Conclusion

This study integrated the metabolomic and transcriptomic analyses of the light-sensitive perilla variety under different light intensity. The light intensity significantly affects the color, metabolite accumulation and gene expression involved in the main anthocyanin biosynthesis in perilla leaves. Based on WGCNA analysis, key genes and transcription factors were identified. What’s more, 6 MYBs and 4 bZIPs were predicted to play important roles in light-regulated anthocyanin biosynthesis. The identified key genes and regulatory factors will help us to understand the potential mechanism of photo-regulated anthocyanin accumulation in perilla.

## Statistical analysis

Statistical analyses were performed by Student’s *t*-test and one-way ANOVA using SPSS 23.0 (SPSS Inc., Chicago, IL, United States). Least significant difference (LSD) was used to compare treatment means, and *p* = 0.05 was considered as statistically significant.

## Data availability statement

The datasets presented in this study are deposited in the Genome Sequence Archive in National Genomics Data Center, Beijing Institute of Genomics, Chinese Academy of Sciences. The accession number is PRJNA837648.

## Author contributions

GX: writing – original draft, conceptualization, methodology, formal analysis, investigation, and data curation. HW: supervision, project administration, and funding acquisition. QS: planned and designed the research, methodology, data curation, and writing – review and editing. XZ and ZL: performed the experiments. HJ: supervision. DW: data analysis. JH and KX: plants cultivation. All authors have read and approved the final version of the manuscript.

## Footnotes

1
www.r-project.org

2
http://www.kegg.jp/kegg/compound/

3
https://www.ncbi.nlm.nih.gov/

4
https://www.ncbi.nlm.nih.gov/refseq/about/nonredundantproteins

5
http://www.geneontology.org/

6
http://www.kegg.jp/kegg/pathway.html

7
http://itol.embl.de/index.shtml
