# Supplementary material for: Integrated metabolomic and transcriptomic analyses reveal molecular response of anthocyanins biosynthesis in perilla to light intensity
Source: Front Plant Sci. 2022 Sep 23;13:976449. doi: 10.3389/fpls.2022.976449 (PMC9540795; doi:10.3389/fpls.2022.976449)
Supplement: Supplementary file 1 [file Data_Sheet_1.docx]

**SUPPLEMENTARY MATERIAL**


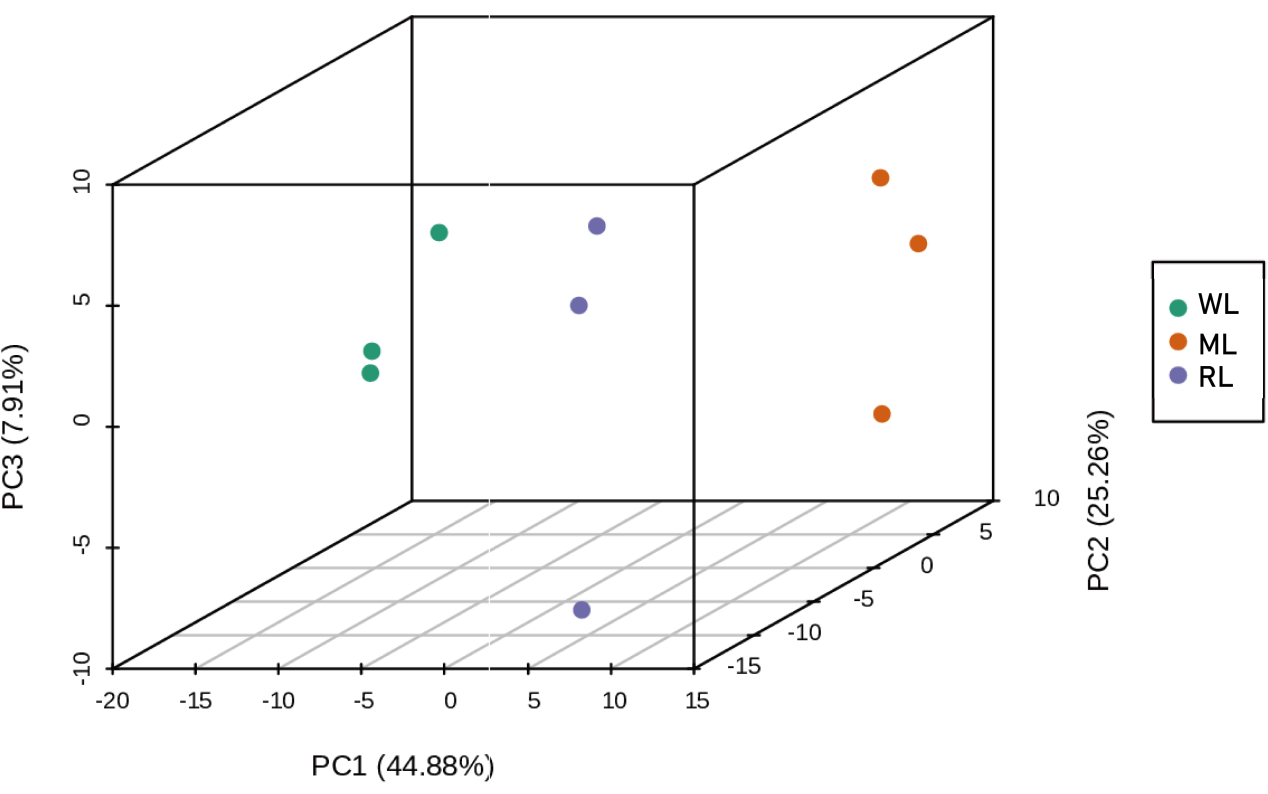


FigureS1. PCA analysis of metabolomic samples.


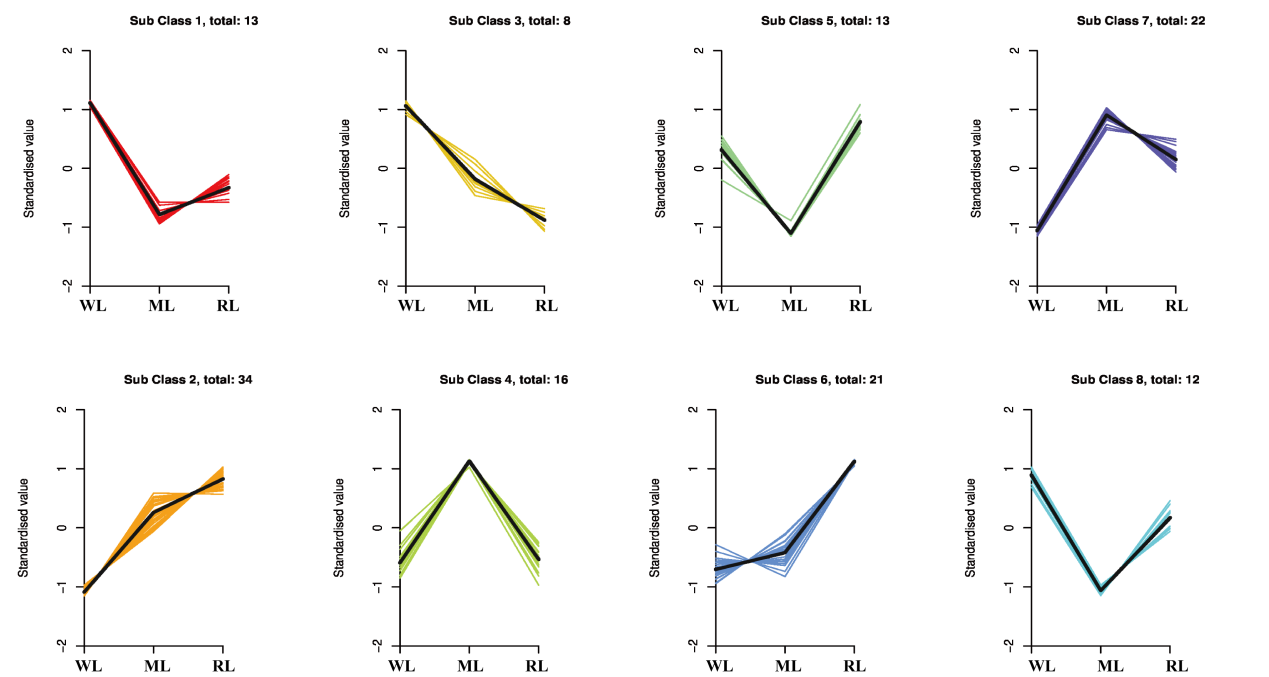


Figure S2. Classification of metabolites according to their expression trends in WL, ML, and RL. Naringin chalcone, Ceninin-3-O -(6" -O-p-coumaryl) glucoside, Malonylshisonin belongs to Subclass7, Dihydroquercetin belongs to Subclass4, Ceninin-3, 5-O-glucoside belongs to Subclass8, and Ceninin-3-O -(6 "-O-P-coumaryl) glucoside-5-O-glucoside belongs to Subclass2.


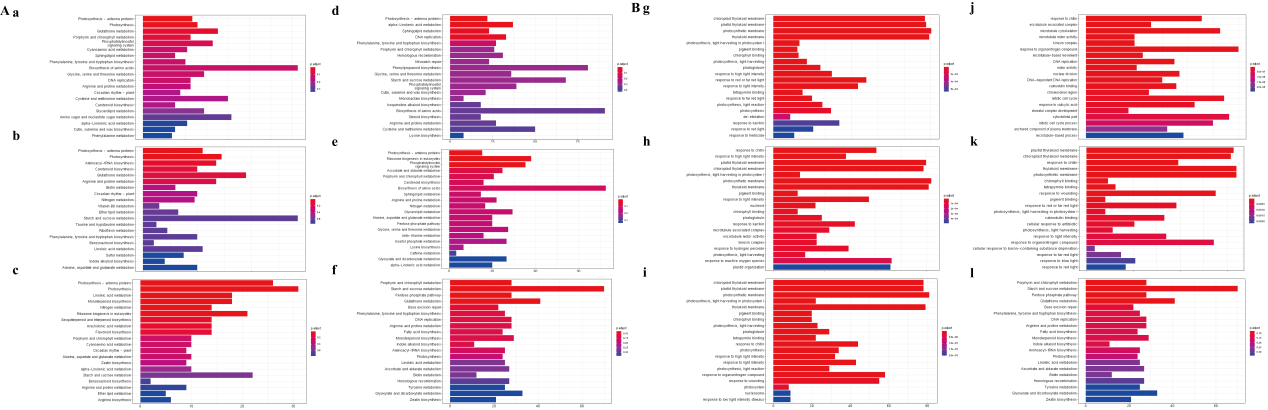


Figure S3. GO and KEGG enrichment of transcriptomic. (a-f) GO enrichment of DEGs in WL vs ML12h, WL vs ML6d, WL vs RL, ML12h vs ML6d, ML12h vs RL, ML6d vs RL. (g-l) KEGG enrichment of DEGs in WL vs ML12h, WL vs ML6d, WL vs RL, ML12h vs ML6d, ML12h vs RL, ML6d vs RL.


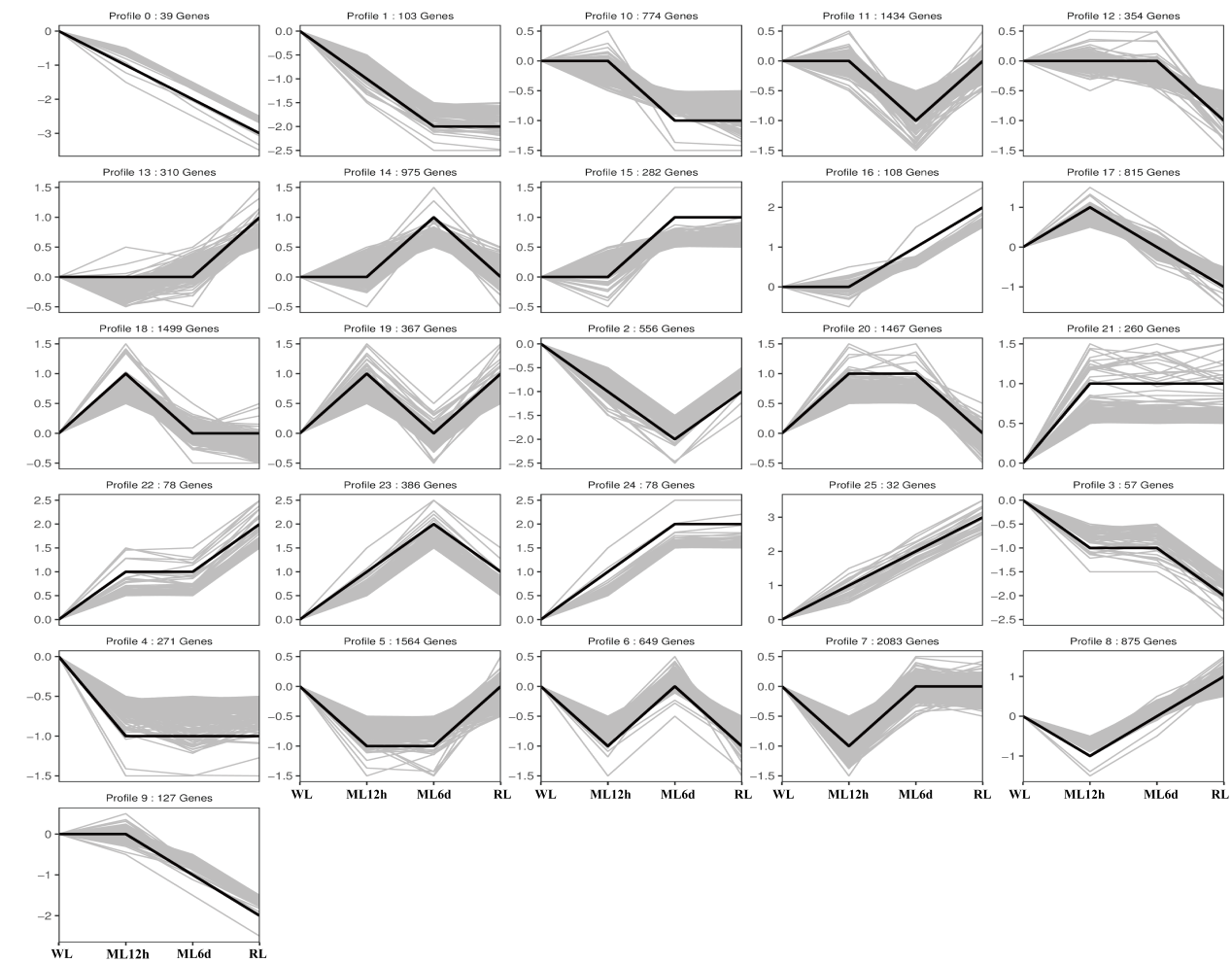


Figure S4. Classification of gene expressions form transcriptomic database based on K-means analysis.


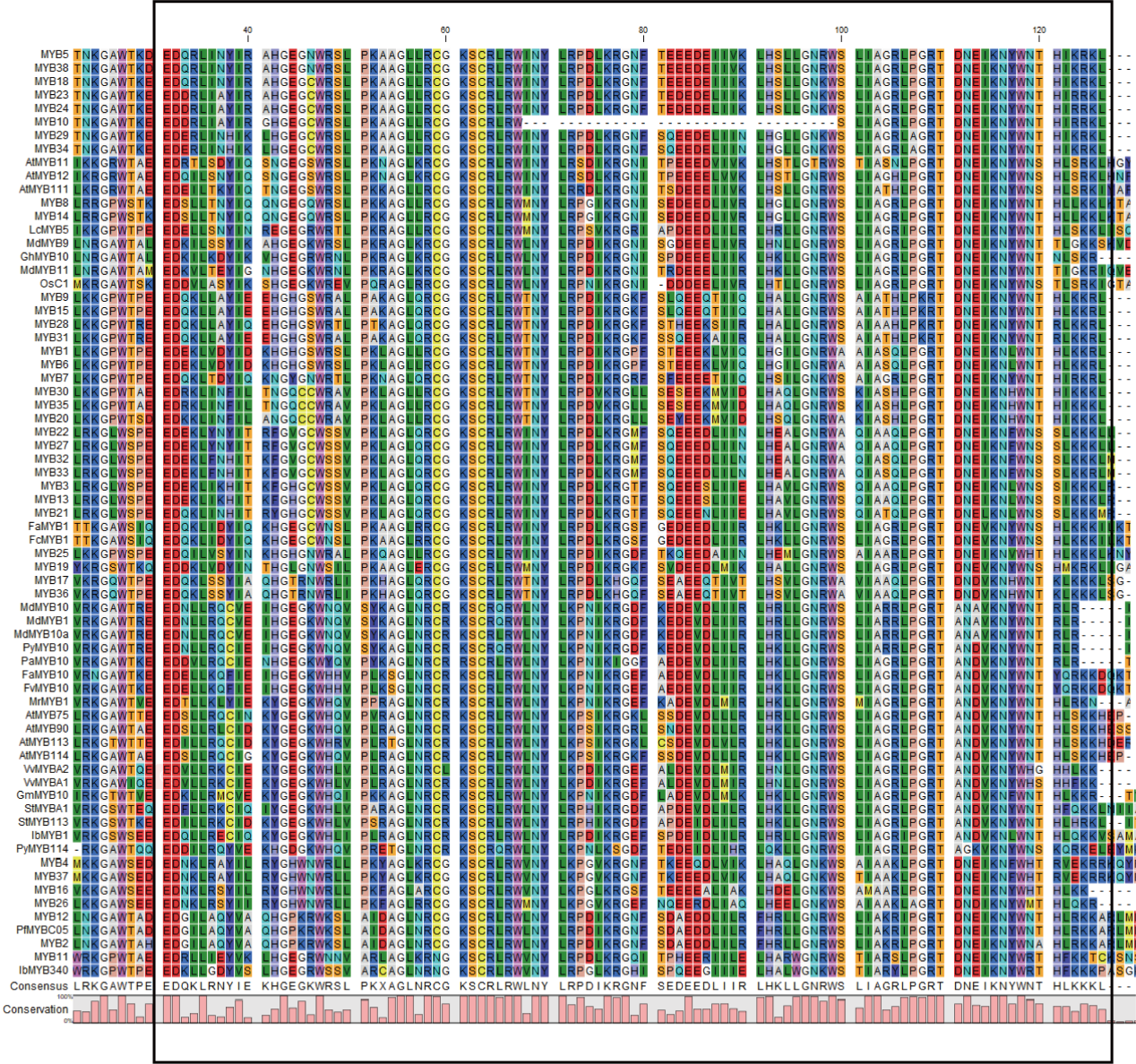


Figure S5. The conserved domain of MYBs involving in phylogenetic relationships.


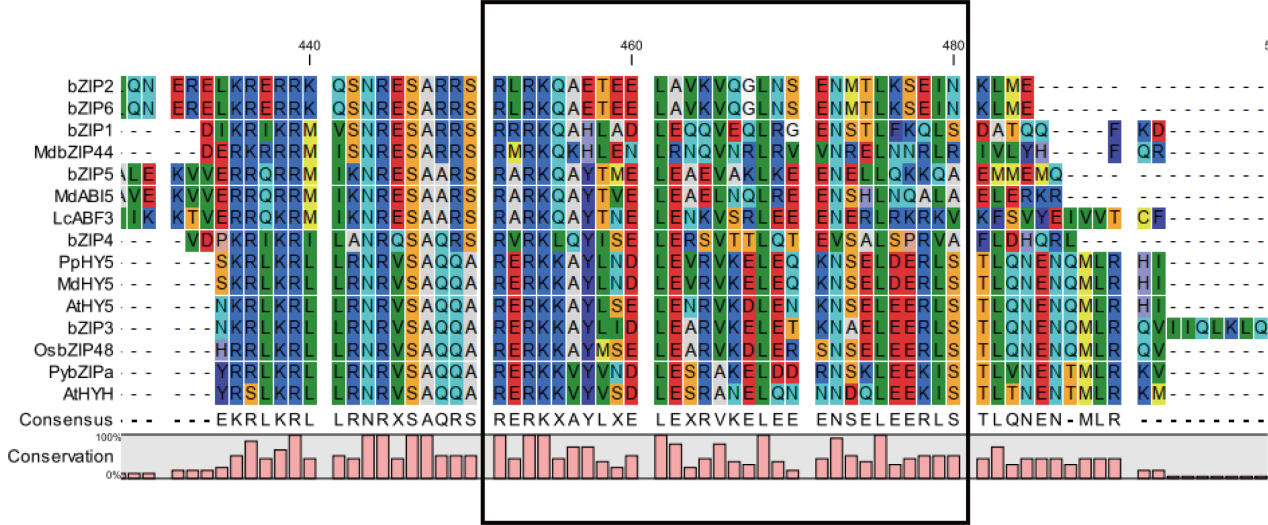


Figure S6. The conserved domain of bZIPs involving in phylogenetic relationships.


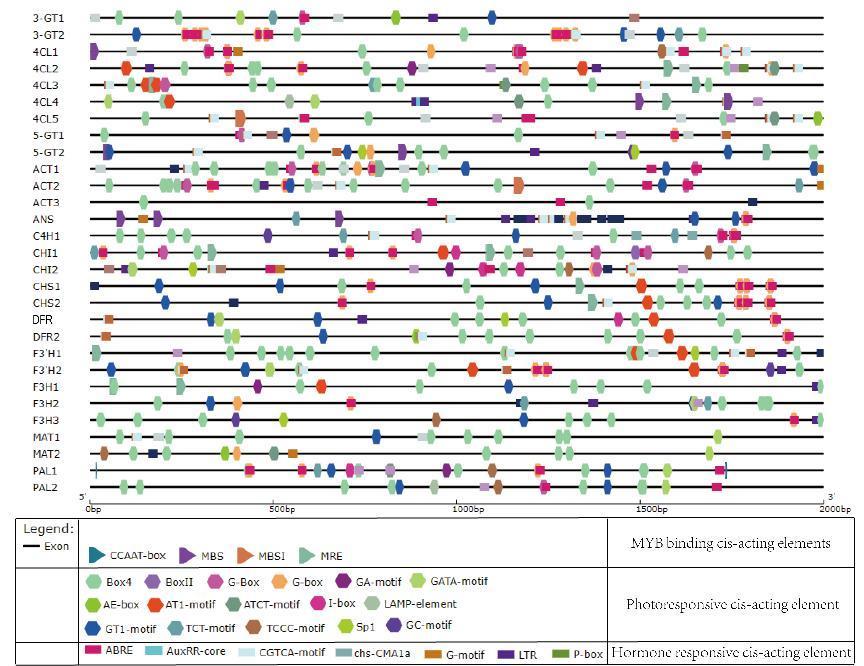


Figure S7. Cis-acting element analysis of 2000bp upstream of structural gene promoter.

Table S1. The type of metabolite in the metabolome

| Meta | Number |
| --- | --- |
| Flavonols | 103 |
| Flavones | 72 |
| Anthocyanidins | 34 |
| Flavanones | 24 |
| Flavonoid carbonoside | 20 |
| Isoflavones | 17 |
| Chalcones | 10 |
| Flavanonols | 6 |
| Flavanols | 6 |
| Biflavones | 1 |

Table S2. The number of differentially expressed metabolite (DEM) in the metabolome

| **Group name** | **All diff** | **Down regulated** | **Up regulated** |
| --- | --- | --- | --- |
| WL vs ML | 90 | 28 | 62 |
| WL vs RL | 55 | 16 | 39 |
| WL vs RL | 81 | 14 | 67 |

| **Sample**  **name** | **Total**  **reads** | **Total**  **bases** | **GC**  **content** | **Q20** | **Q30** | **Unique**  **mapped %** | **Multiple**  **mapped%** | **Unmapped%** |
| --- | --- | --- | --- | --- | --- | --- | --- | --- |
| WL1 | 48,465,434 | 7,269,815,100 | 48.81% | 97.58% | 93.16% | 82.94 | 10.19 | 6.87 |
| WL2 | 72,243,630 | 10,836,544,500 | 48.95% | 97.49% | 93.01% | 83.33 | 9.76 | 6.91 |
| WL3 | 59,017,794 | 8,852,669,100 | 49.23% | 97.40% | 92.78% | 81.42 | 10.09 | 8.49 |
| ML12h1 | 51,651,414 | 7,747,712,100 | 48.24% | 97.48% | 92.91% | 80.81 | 10.17 | 9.02 |
| ML12h2 | 55,027,026 | 8,254,053,900 | 47.97% | 97.54% | 93.08% | 78.55 | 11.36 | 10.09 |
| ML12h3 | 73,528,134 | 11,029,220,100 | 48.21% | 97.54% | 93.06% | 79.9 | 10.38 | 9.72 |
| ML6d1 | 54,155,966 | 8,123,394,900 | 48.58% | 97.63% | 93.29% | 81.64 | 9.81 | 8.55 |
| ML6d 2 | 60,080,002 | 9,012,000,300 | 48.06% | 97.71% | 93.42% | 85.09 | 9.22 | 5.69 |
| ML6d 3 | 64,995,912 | 9,749,386,800 | 48.22% | 97.62% | 93.29% | 77.86 | 12 | 10.14 |
| RL1 | 52,863,102 | 7,929,465,300 | 48.33% | 97.54% | 93.11% | 82.84 | 10.4 | 6.76 |
| RL 2 | 55,495,160 | 8,324,274,000 | 47.68% | 97.51% | 92.99% | 82.85 | 10.32 | 6.83 |
| RL 3 | 54,098,858 | 8,114,828,700 | 47.96% | 97.54% | 93.09% | 81.566 | 10.336 | 8.097 |
| Total &  Average | 701,622,432 | 105,243,364,800 | 48.35% | 97.55% | 93.10% | 82.94 | 10.19 | 6.87 |

Table S4. Transcriptome data quality.

Table S5. Number of genes encoding flavonoids and anthocyanin pathway synthase in *Perilla* genome.

| Enzymes | Numbers | Enzymes | Numbers |
| --- | --- | --- | --- |
| PAL | 6 | ANS | 1 |
| C4H | 8 | 3-GT | 2 |
| 4CL | 20 | 5-GT | 10 |
| CHS | 13 | ACT | 12 |
| CHI | 3 | MAT | 10 |
| F3H | 5 | F6H | 3 |
| F3'H | 4 | UBGT | 6 |
| DFR | 2 | FNSⅡ | 13 |

Table S8. Accessions of homologous MYBs and bZIPs from other species.

| Protein | Accession Numbers | Species |
| --- | --- | --- |
| VvMYBA2 | BAD18978.1 | Vitis vinifera |
| VvMYBA1 | BAD18977.1 | Vitis vinifera |
| StMYBA1 | ALA13582.1 | Solanum tuberosum |
| StMYB113 | ALA13583.1 | Solanum tuberosum |
| PbMYB114 | ASY06612.1 | Pyrus x bretschneideri |
| OsC1 | BAF19004.1 | Oryza sativa Japonica |
| PfMYBC05 | BAC77066.1 | Perilla frutescens var. crispa |
| PaMYB10 | ALH21137.1 | Prunus avium |
| PyMYB10 | ADN26574.1 | Pyrus pyrifolia var. culta |
| MrMYB1 | ADG21957.1 | Morella rubra |
| MdMYB11 | AAZ20431.1 | Malus domestica |
| MdMYB10a | ABB84755.1 | Malus domestica |
| MdMYB9 | ABB84757.1 | Malus domestica |
| MdMYB10 | ACQ45201.1 | Malus domestica |
| MdMYB1 | ADQ27443.1 | Malus domestica |
| LcMYB5 | QRV61382.1 | Litchi chinensis |
| LcMYB1 | APP94121.1 | Litchi chinensis |
| IbMYB340 | QGP74108.1 | Ipomoea batatas |
| IbMYB1 | BAG68211.1 | Ipomoea batatas |
| GhMYB10 | AAK19615.1 | Gossypium hirsutum |
| GmMYB10 | ACM62751.1 | Garcinia mangostana |
| FaMYB10 | QIZ03070.1 | Fragaria x ananassa |
| FaMYB1 | QIZ03071.1 | Fragaria x ananassa |
| FvMYB10 | ABX79948.1 | Fragaria vesca |
| FcMYB1 | ADK56163.1 | Fragaria chiloensis |
| AtMYB114 | AT1G66380.1 | Arabidopsis thaliana |
| AtMYB113 | AT1G66370.1 | Arabidopsis thaliana |
| AtMYB111 | AT5G49330.1 | Arabidopsis thaliana |
| AtMYB90 | AT1G66390.1 | Arabidopsis thaliana |
| AtMYB75 | AT1G56650.1 | Arabidopsis thaliana |
| AtMYB12 | AT2G47460.1 | Arabidopsis thaliana |
| AtMYB11 | AT3G62610.1 | Arabidopsis thaliana |
| PyHY5 | QRR19189.1 | Pyrus pyrifolia |
| OsbZIP48 | LOC_Os06g39960 | Oryza sativa Japonica |
| MdHY5 | NP_001280752.1 | Malus domestica |
| MdbZIP44 | XP_008378822.2 | Malus domestica |
| MdABI5 | XP_028946642.1 | Malus domestica |
| LcABF3 | AYM94134.1 | Litchi chinensis |
| AtHYH | AT3G17609.1 | Arabidopsis thaliana |
| AtHY5 | AT5G11260.1. | Arabidopsis thaliana |

Table S9. qRT-PCR primers for candidate genes.

| Genes | Primers |
| --- | --- |
| *CHI1* | CHI1-F: CTGGAAAGCAGTTGGGAAATA  CHI1-R: TTGGAGAAGGCAATCGTGAG |
| *CHI2* | CHI2-F: GGAAAGCAGTTGGGAAATACG  CHI2-R: CTTTGGAGAAGGCAATCGTG |
| *ANS* | ANS-F: GGTGGTGCGGAGGATCTAAT  ANS-R: GTGGAGGATGAAGGTGAGGG |
| *DFR* | DFR-F: TATTCTTGTTTGAGCACCCTG  DFR-R: CCCATATCCACCAATTTCTT |
| *MYB-related2* | PfMYB-r2-F: TCCAAGGTTGTGCGTGAGTC  PfMYB-r2-R: GCTTACGAGGATACGGGTGC |
| *MYB-related4* | PfMYB-r4-F: ATTCCTAATGCCTGAGACCG  PfMYB-r4-R: AGATTGATCCGAACCCAACG |
| *bZIP2* | PfbZIP2-F: AATTTGACGGGCTTGCTATA  PfbZIP2-R: AACTCCGTTACTTCCATCACTA |
| *bZIP6* | PfbZIP6-F: TAAATTCCGAGAATATGACCC  PfbZIP6-R: TGTCCTCGTTCCTAACATCG |
| *bHLH1* | PfbHLH1-F: CCAATCGCAGTAATCCAGTC  PfbHLH1-R: TTCAGGAGGAGATGAACCCT |
| *bHLH7* | PfbHLH7-F: TTCCAGGTGTAATAACGAAGTGA  PfbHLH7-R: GATCGAGATGACGGTTGAGG |
| *MYB4* | PfMYB4-F: CGCTGTTTGAAGAGGAGTTT  PfMYB4-R: ATCACTGCTTTCGCATTCTG |
| *MYB8* | PfMYB8-F: AAATCGCCACCAAGCCCAAGA  PfMYB8-R: CGCCGTCACCCTCACCTTCT |
| *MYB12* | PfMYB12-F: GCAATCTCAGTCACAGCAGCAA  PfMYB12-R: GACCCAATCCAATCCAGACG |
| *MYB14* | PfMYB14-F: CAGGTCGAACGGACAATGAG  PfMYB14-R: TCTTGGGCTTGGTGGAGATT |
| *MYB37* | PfMYB37-F: CGCTGTTTGAAGAGGAGTTT  PfMYB37-R: ATCACTGCTTTCGCATTCTG |
